# P79 Fluoroquinolone fear: when safety collides with stewardship

**DOI:** 10.1093/jacamr/dlaf230.086

**Published:** 2025-12-04

**Authors:** Tamara Hamoodi, Matthew Huntley, Annabel Healey

**Affiliations:** Croydon Health Services, Croydon, UK; Croydon Health Services, Croydon, UK; Croydon Health Services, Croydon, UK

## Abstract

**Background:**

In 2024 the Medicines and Healthcare products Regulatory Agency (MHRA) updated on their previous alerts and recommended further restrictions on the use of fluoroquinolones (FQ). While intended to protect patient safety, such alerts risk being interpreted as blanket prohibitions. Where these antibiotics remain critical options, particularly for resistant or complex infections, risk communication must be balanced with stewardship strategies that preserve clinical confidence and enable appropriate use.

**Objectives:**

To explore perceptions of FQ use post MHRA alert among prescribers, pharmacists and microbiologists at Croydon University Hospital (CUH), and to identify behavioural impacts, barriers and enablers to safe and confident prescribing.

**Methods:**

An anonymous, cross-sectional survey was distributed in June 2025 to clinicians, pharmacists and microbiology teams at CUH. The survey included structured Likert-scale and free-text questions exploring awareness of the MHRA alert, confidence in assessing risks, influence on prescribing behaviour, sources of safety information, perceived support from AMS services and local prescribing culture.

**Results:**

Twenty-four responses were collected. While 75% of respondents were aware of the MHRA alert, only 33% felt ‘very confident’ describing associated risks. Nearly 40% reported reduced confidence in prescribing FQs since the alert. A minority (4/24) described withholding a FQ in a scenario where they felt it was clinically appropriate due to safety concerns. Respondents received information from a range of sources including AMS teams, the BNF, emails and local guidelines. Support from pharmacy and microbiology was rated variably with just over 70% of respondents citing they received helpful support regarding FQ use, however several pharmacists reporting feeling uncertain or unsupported, highlighting that knowledge alone may not translate into confidence.

**Conclusions:**

MHRA safety communication has improved awareness but introduced therapeutic hesitancy in some clinicians. These findings support the need for AMS approaches that actively reinforce safe, supported prescribing, rather than defaulting to avoidance. FQs should be framed as high-risk, high- value agents requiring informed, case-based decisions. AMS teams play a vital role in shaping this narrative through education, reassurance and cultural leadership. Looking forward, embedding clinical decision support into digital systems, including AI-driven risk-benefit prompts offers a promising tool to sustain confident, safe prescribing at the point of care.

